# Transcatheter tricuspid interventions: time to re-think guidelines?

**DOI:** 10.18632/aging.102805

**Published:** 2020-01-27

**Authors:** Ana Paula Tagliari, Maurizio Taramasso

**Affiliations:** 1 Cardiac Surgery Department, University Hospital of Zurich University of Zurich, Zurich, Switzerland; 2 Postgraduate Program in Health Sciences: Cardiology and Cardiovascular Sciences, Faculdade de Medicina da Universidade Federal do Rio Grande do Sul, Porto Alegre, Brazil

**Keywords:** tricuspid valve, tricuspid regurgitation, transcatheter tricuspid valve interventions

Tricuspid regurgitation (TR) is a heterogeneous disorder defined by the backflow of blood from the right ventricle into the right atrium, what in normal conditions should be avoided by systolic leaflets coaptation. In almost 90% of TR cases, the valve is anatomically preserved, and the regurgitation is secondary to right ventricular or right atrial dilation (functional TR) due to left-sided heart disease, atrial fibrillation, or pulmonary hypertension, culminating in tricuspid annular dilation or leaflet tethering.

The presence of some TR degree is considered the most common valvular heart disease, affecting from 65 to 85% of the population [1], what means a 1.6 and 3.0 million individuals prevalence, and a 200.000 and 300.000 yearly incidence in the United States and Europe, respectively [2]. If considered only significant TR (moderate/severe), the prevalence is one in 25 individuals with 75 years old and over, similar to that observed in significant aortic stenosis [3], and probably underestimated, owing to its long clinically silent time (no specific murmur or symptoms). Still regarding age, it is remarkable that this variable is not only a predictor of TR, but it is also associated with worse outcomes and increased TR recurrence after surgical treatment [4].

Despite its high prevalence and the implied two-fold increase in mortality [5], more than 90% of the patients with significant TR will never receive surgical management [2,6], the only treatment considered class I recommendation in the current guidelines. Two main reasons are responsible for this undertreatment. The first is an obsolete belief that functional TR should improve or disappear after the primary left-sided problem treatment, which was refuted by well-conducted studies proving that TR remains or progresses after left-sided interventions [7]. The second is an unacceptable high morbimortality rate associated with conventional TR surgery, what, unfortunately, remains at least partially true, since TR carries the highest surgical mortality among all cardiac valve surgeries (8.8% – 9.7%) [4]. However, such a bad reputation is in part biased by the advanced stage that patients are referred to surgery, with severe right ventricle dysfunction and end-organ damage, totally opposed to left-sided valves, where surgery is performed before the onset of left ventricle dysfunction or symptoms.

Efforts to change the paradigm of poor outcomes related to TR management, and to reduce the gap between the number of affected and the number of treated patients, make the engagement of the “forgotten” valve in the transcatheter intervention era an urgent necessity, and a new field for device development and clinical research.

In this scenario, the recent paper published by Taramasso et al. [8] “Transcatheter versus medical treatment of symptomatic severe tricuspid regurgitation” brings some scientific light over a relatively unexplored issue.

In this propensity-matched case-control study the treated group was derived from the TriValve (Transcatheter Tricuspid Valve Therapies) registry, the largest multicenter, multidevice cohort of patients submitted to transcatheter therapies; while the control was gathered from two large retrospective registries in Europe and North America, providing a high degree of reliability and validity to the study.

Summarizing the main outcomes, the authors showed that transcatheter tricuspid valve interventions (TTVI) was associated with lower rates of 1-year rehospitalization due to heart failure (26±3% vs. 47±3%; *p*<0.0001), as well as reduced 1-year mortality (23±3% vs. 36±3%; *p*=0.001), and the composite endpoint of death or heart failure rehospitalization (32±4% vs. 49±3%; *p*=0.0003). The composite endpoint benefit remained statistically significant even after adjusting for sex, New York Heart Association (NYHA) class, right ventricular dysfunction, and atrial fibrillation (HR 0.39, 95% CI 0.26 – 0.59; *p*<0.0001). A similar magnitude of effect could be observed after further adjustment for mitral regurgitation and pacemaker/defibrillator implanted (HR 0.35, 95% CI 0.23 – 0.54; *p*<0.0001).

Another notable observation was that in those patients who hadn’t a substantial TR reduction after TTVI, the outcomes were similar to the control group, indicating that TR reduction significantly impacted outcomes (heart failure rehospitalization: 41.8% vs. 45.9%; 1-year mortality: 27% vs. 35%).

Regardless the diversity of devices utilized in the study, procedural success, defined as patient alive at the end of the procedure, with device successfully implanted, delivery system retrieved, and residual TR <3+, occurred in 86% of patients and did not differ between the adopted approaches (*p*=0.8).

To realize the importance of this study in the present era, we need first to understand that TR is far from being merely a marker of concurrent cardiac disorders. Instead of this, TR is a real public health crisis, affecting millions of patients, leading to severe heart failure and excess mortality and treated only in a minimal fraction of affected patients [6].

In view of the lack of randomized clinical trials evaluating the role of TTVI in symptomatic severe TR patients, especially elderly, this paper constitutes pioneer evidence supporting a more interventional approach, suggesting that it could be the time to change current guidelines and to include TTVI in the role of TR treatment options.

## References

[1] Singh JP, et al. Am J Cardiol. 1999; 83:897–902. 10.1016/S0002-9149(98)01064-9 10190406

[2] Stuge O, Liddicoat J. J Thorac Cardiovasc Surg. 2006; 132:1258–61. 10.1016/j.jtcvs.2006.08.049 17140937

[3] Topilsky Y, et al. JACC Cardiovasc Imaging. 2019; 12:433–42. 10.1016/j.jcmg.2018.06.014 30121261

[4] Zack CJ, et al. J Am Coll Cardiol. 2017; 70:2953–60. 10.1016/j.jacc.2017.10.039 29241483

[5] Wang N, et al. Eur Heart J. 2019; 40:476–84. 10.1093/eurheartj/ehy641 30351406

[6] Enriquez-Sarano M, et al. Prog Cardiovasc Dis. 2019; 19:30134–3. 10.1016/j.pcad.2019.10.009 31707061

[7] Pfannmueller B, et al. Thorac Cardiovasc Surg. 2013; 61:386–91. 10.1055/s-0033-1333844 23475798

[8] Taramasso M, et al. J Am Coll Cardiol. 2019; 74:2998–3008. 10.1016/j.jacc.2019.09.028 31568868

